# Supplementary material for: MatSwarm: trusted swarm transfer learning driven materials computation for secure big data sharing
Source: Nat Commun. 2024 Oct 28;15:9290. doi: 10.1038/s41467-024-53431-x (PMC11519480; doi:10.1038/s41467-024-53431-x)
Supplement: Supplementary file 1 — Supplementary Information [file 41467_2024_53431_MOESM1_ESM.pdf]

SUPPLEMENTARY INFORMATION

MatSwarm: Trusted Swarm Transfer Learning Driven Materials Computation for Secure Big Data Sharing

Ran Wang[1,2,3], Cheng Xu✉[1,2,4*], Shuhao Zhang[3], Fangwen Ye[1], Yusen Tang[1], Sisui Tang[1], Hangning Zhang[1], Wendi Du[1] and Xiaotong Zhang✉[1,2,4*]

[1*]School of Computer and Communication Engineering, University of Science and Technology Beijing, 100083, Beijing, China.
[2*]Beijing Advanced Innovation Center for Materials Genome Engineering, University of Science and Technology Beijing, 100083, Beijing, China.
[3]College of Computing and Data Science, Nanyang Technological University, 639798, Singapore.
[4*]Shunde Innovation School, University of Science and Technology Beijing, 528399, Guangdong, China.

*Corresponding author(s). E-mail(s): xucheng@ustb.edu.cn; zxt@ies.ustb.edu.cn;

Supplementary Note 1

Related Work

Federated Learning over Non-I.I.D. Data

Federated learning aims to train a global model that can learn from data distributed across different devices while preserving data privacy [1]. Although FL offers a promising privacy-preserving approach, numerous challenges arise when applied in real-world scenarios [2]. One of the significant issues is the decline in model accuracy and generalization due to non-independent and identically distributed (non-i.i.d.) data [3]. To tackle the FL problem with non-i.i.d. data, McMahan et al. [4] first proposed the Federated Averaging (FedAvg) algorithm. While they claimed that FedAvg could handle non-i.i.d. data to some extent, the algorithm itself struggles with model divergence caused by non-i.i.d. data, especially when using complex models like neural networks in FL [3].

Data-based Methods. Building on the FedAvg algorithm, many researchers have proposed various optimization solutions to address these issues [5–7]. Intuitively, the performance degradation in FL due to non-i.i.d. data stems from heterogeneous data distribution, so data-based methods aim to resolve this by modifying the distribution. Data sharing and augmentation are two primary solutions currently employed. Data sharing [7] is straightforward and effective for handling non-i.i.d. data in horizontal FL. However, it has significant drawbacks: obtaining a truly uniformly distributed global dataset is challenging, and downloading part of the global dataset to each client for model training contradicts the principles of privacy-preserving learning. Initially, data augmentation [8–10] was a technique to enhance training data diversity through random transformations or knowledge transfer and could mitigate local data imbalance in FL, mainly applied in classification problems. However, most of these techniques rely on global dataset sharing, potentially increasing the risk of data privacy leakage.

Penalty Terms-based Methods. Given the privacy risks associated with data-based methods, some researchers have introduced penalty terms in the objective function to address the non-i.i.d. problem. Li et al.[6] proposed the FedProx algorithm, which adds a penalty term to the local objective function in FedAvg to reduce the bias between local models and the global model, thus mitigating the impact of data heterogeneity on model training. Similarly, Karimireddy et al. [11] introduced the SCAFFOLD (Stochastic Controlled Averaging for Federated Learning) algorithm, which corrects client drift by introducing control variates in local updates, addressing data heterogeneity. FedDC [12] tackles the non-i.i.d. problem by focusing on local drift decoupling and correction, adding a gradient correction term inspired by methods like SCAFFOLD to further reduce local gradient variance. However, methods that introduce penalty terms often require additional information exchange between the server and clients, increasing communication overhead in FL environments.

Knowledge Distillation-based Methods. To reduce communication overhead, knowledge distillation is also a promising idea for personalized federated learning

[13]. Knowledge distillation helps align local models more closely with the global model, thus improving overall performance. The concept of transferring information from larger models to smaller ones was first proposed by Bucilua et al. [14] and later popularized as knowledge distillation by Hinton et al. The primary motivation in FL is to transfer knowledge from the server or other clients to a specific client, enhancing its performance on unknown heterogeneous data. Zhang et al. [15] proposed a data-free knowledge distillation method, which explores the input space of local models through a generator, transfers local model knowledge to the global model, and employs hard sample mining strategies for effective knowledge distillation, thereby alleviating the performance degradation caused by direct model aggregation. Nonetheless, current knowledge distillation methods mainly apply to classification problems, and there is no effective solution for the regression problems prevalent in the field of materials science.

Secure Collaborative Computing based on Blockchain

Emerging blockchain technologies offer a decentralized solution that promotes trusted interaction between data providers and consumers [16]. For example, Chen et al. [17] proposed a blockchain-based medical data information system, where data management and access control are performed through a licensed blockchain architecture. Fan et al. [18] proposed a secure one-to-many data-sharing mechanism for the Internet of Vehicles. These solutions provide a distributed framework and a trusted environment for data sharing without the need for a third party. However, the security of sensitive data sharing cannot be fully guaranteed, as transaction data on blockchain is open and transparent to all participants, posing potential security risks.

Blockchain enabled Federated Learning. In recent years, swarm learning has been proposed by integrating distributed machine learning and blockchain to address the security of raw data during data sharing. The combination of blockchain and FL is one of the typical solutions [19–21]. These approaches maintain control over shared raw data and improve the anti-attack capabilities of models during the FL process. For instance, Pokhrel et al. [22] proposed a blockchain-based autonomous federated learning design for privacy awareness and efficient vehicle network communication, although the high computation cost increases overall delay time. Warnat et al. [23] used swarm learning to develop disease classifiers using distributed data, focusing on four use cases of heterogeneous diseases (COVID-19, tuberculosis, leukemia, and lung pathologies). Saldanha et al. [24] have demonstrated the successful use of swarm learning on large, multicentric datasets of gigapixel histopathology images from over 5,000 patients. However, the models in this scheme are stored in blockchain nodes and are not fully encrypted, making them vulnerable to tampering attacks. Although combining blockchain with FL has significant potential, the issue of parameter leakage in FL models, which can expose raw data, must be urgently addressed.

Privacy Computing in Federated Learning. To address above-mentioned issue, researchers have proposed various privacy computing techniques that can be used with FL [25, 26]. Wei et al. [27] developed a new privacy framework based on differential privacy, although it may reduce model prediction accuracy and efficiency.

To overcome this limitation, Liu et al. [28] proposed using Additive Homomorphic Encryption (AHE) for multi-party computation of neural networks in federated transfer learning, ensuring the security of model parameters while preserving accuracy and efficiency. However, AHE is only suitable for federated transfer learning based on neural network algorithms, limiting its applicability. Cryptographic algorithms [29] can also be used to prevent sensitive data leakage, but they can reduce the accuracy of FL model training due to their complexity. Therefore, researchers must balance privacy concerns and computational efficiency when selecting a privacy-preserving method for FL. One promising option is a Trusted Execution Environment (TEE) [30], a hardware-based privacy computing technology that provides complete privacy protection calculations while ensuring the confidentiality and integrity of the code and data loaded in the TEE.

Supplementary Table 1 summarizes existing secure collaborative computing frameworks, analyzing their research gaps and discussing the advantages of our proposed framework. As shown, current research faces specific problems regarding security, efficiency, and model accuracy. Therefore, we propose the *MatSwarm* framework, which addresses significant issues in existing research and offers new solutions for collaborative computing in material science.

Supplementary Note 2

The General Introduction of NMDMS

`MatSwarm` introduces novel collaborative computing concepts and methodologies to advance research and development in materials science, particularly in material performance prediction, leveraging the National Material Data Management and Services (NMDMS) Platform, as illustrated in Supplementary Fig. 1. Employed in the National Materials Genome Engineering (MGE) project in China, NMDMS has accumulated **over 14 million** pieces of valid material data. This secure big-data sharing platform provides foundational support for MGE data applications, offering data consumers access to a vast collection of material data resources from more than thirty research institutions across China. The platform addresses key challenges in collecting, storing, and utilizing multi-source material data, thereby enhancing data utilization, promoting service sharing, accelerating material discovery, and meeting the demands for high-throughput calculations and experiments. It plays a crucial role in accelerating the development of materials science.

Various materials organizations fully utilize the data resources of the NMDMS Platform, leveraging the advantages of big data to achieve data-driven technological innovation in materials science. Relevant case studies include the machine learning-assisted multi-performance optimization of new cobalt-based superalloys, rational design of solution-strengthened copper alloys based on key elemental characteristics, adaptive optimization design of high-hardness high-entropy alloys, and the construction and composition optimization of high-dimensional phase diagrams for ferroelectric materials. Future efforts will focus on achieving collaborative modeling among various materials organizations, further improving the collaborative computational services provided by the platform to meet the application requirements for secure sharing of materials big data, and accelerating the research and development process in material science.

In constructing a secure data-sharing platform for materials genome engineering, the NMDMS platform has incorporated the blockchain, proposing solutions for data collection, storage, usage, and security. By building a blockchain architecture on the underlying MongoDB database and using a method where transaction data is stored on-chain and original data off-chain, centralized management and security auditing of different types of databases are achieved. This method prevents data leakage and tampering and ensures traceability. Full-text retrieval of multi-source heterogeneous data is achieved through methods such as inverted indexing and Merkle Patricia Trees (MPT). Through federated learning and secure multi-party computation, multi-party collaborative prediction, modeling, and discovery of materials data are realized. The NMDMS Platform enhances data utilization, promotes the sharing process of materials data, accelerates materials discovery, and supports the data needs of high-throughput computation and high-throughput experiments, ultimately aiding the design of new materials.

The System Architecture of NMDMS

The framework of the National Materials Data Management and Services (NMDMS) platform [31] includes data providers, data consumers, data service providers, and the core blockchain framework, which provides hub services connecting these components.

1) **Platform Participants:** The NMDMS platform for material genome engineering comprises data providers, data consumers, and data service providers. Any organization within the NMDMS platform can serve as a data provider or data consumer. Data providers contribute data sources to the platform, interacting with it through the blockchain framework. Original data can be uploaded to the platform's uniform storage system or stored locally at the data provider's site. Data consumers initiate access or service requests for shared data within the platform, also interacting through the blockchain framework. The blockchain framework records all transactions among data providers, data consumers, and data service providers. Data service providers offer essential services to data providers and consumers via internal/external APIs embedded in the blockchain, enabling authorized users to share material data on the platform and collaboratively retrieve and analyze material data.

2) **Blockchain Framework:** The blockchain serves as middleware in the entire platform architecture. Data providers and consumers, as nodes on the blockchain, send all transaction requests via the external API and issue transaction tasks to various systems within the platform through the internal API. Users do not need to understand the underlying architecture and business processes of the data service provider. The platform's collection, storage, and service systems remain transparent to users. The endorsement node of the blockchain executes the smart contract, with the internal API transferring relevant parameters of the transaction proposal to the platform's subsystems. Subsequently, the endorsement node returns the signature endorsement and proposal execution results to the data provider or consumer. Finally, all transaction results are generated into blocks and synchronized across the entire blockchain via the consensus mechanism, ensuring that all network participants agree on the valid state of the blockchain data, enhancing security and maintaining the integrity and consistency of the distributed ledger. The proposed blockchain framework benefits users by reducing cognitive load and learning costs, while providing a general solution that increases the scalability of the blockchain and serves as a reference for big-data sharing platforms in other industries or domains.

3) **Data Service Provider:** The data service provider encompasses the data collecting, storage, and service subsystems, offering data lifecycle services. In the data collecting subsystem, the data ingestor receives uploaded data and uses the container schema designer to customize the schema representing the original dataset, adhering to the standard data format adopted by the platform. The data storage subsystem stores the original data parsed by the collecting subsystem into different databases and provides the required formatted data to the data consumer and each framework of the data service subsystem. The data service subsystem offers essential data retrieval, multiparty collaborative computing, third-party integration functions, and other services for data consumers. The service result, i.e., the reorganized dataset, is stored in the platform data storage system, and the summary information of the service

result is stored on the blockchain for subsequent sharing. This bidirectional data flow between the data computation and storage system constitutes a virtuous cycle of data and service sharing.

The Platform Function Analysis

Data Collecting Subsystem: The Dynamic Container Model [31, 32] is central to the NMDMS for MGE, and its performance largely determines the overall platform efficiency. To enhance usability, the platform includes a container schema designer that allows users to intuitively modify existing schemas or create entirely new ones using built-in types. The figure shows how the container schema designer's graphical user interface (GUI) can describe the properties and structure of data. This flexibility in creating container schemas improves data normalization quality, making it easier for users to discover and use data. Additionally, the data collecting subsystem provides specialized tools for each data category, offering appropriate operational granularity. These tools enable data collecting from providers and automatic normalization into containerized data sets, reducing user workload. The platform also includes a schema evaluator to assess schema quality. Experts in materials and schemas can correct inappropriate terms and structures, and approved patterns are published on the platform.

Data Storage Subsystem: For data storage, the platform accepts data uploads via web pages or files. Upon entering the data upload page, users can select an existing template or create a new one before uploading data. The data submission format can be web pages or files, including EXCEL, JSON, or XML. When submitting via web pages, users click "Submit via the web page," fill in the metadata and related information, and click "Submit." The transaction record of the uploaded data, including the hash value of the submitted metadata, is stored on the blockchain. The original data directly interacts with the underlying database for storage. Supplementary Fig. 2 illustrates the interface for data upload functions.

Data Service Subsystem: In the data service subsystem, the fundamental service mainly includes the retrieval service, which offers three modes: primary, container-based advanced, and full-text, allowing users to perform complex data queries. In the primary retrieval mode, users can quickly locate datasets through metadata information such as data title, abstract, owner, and keywords. The container-based advanced retrieval mode enables users to impose constraints on data properties of interest to access specific datasets. The full-text retrieval mode allows users to obtain datasets containing multiple keywords in metadata or properties. Each dataset in the retrieval results is represented by a visual interface generated by the corresponding schema.

In terms of integrated service, the computing and analysis tools can be directly integrated into the service module of the platform through a third-party online service interface and transmitted data via an access portal and dedicated API in the service gateway. The framework has integrated several services developed by collaborating teams, such as MatCloud for HTC, OCPMDM for data mining, and an interatomic potential database for atomic simulation. Additionally, based on modules such as

federated learning and secure multi-party computing, multi-party collaborative services such as multi-party joint prediction of material properties and generation of new materials are provided. The platform opens up related services of multi-party collaborative computing to all registered researchers in the materials community, allowing for the development and integration of valuable tools to improve data utilization. This approach ensures the security of material data, promotes the sharing process, and accelerates material discovery.

With the integration of the `MatSwarm` framework into the NMDMS platform, registered organizations will gain access to a broader range of collaborative computing services, significantly enhancing model accuracy, generalization capabilities, and security. In the future, `MatSwarm` will undergo continuous improvement and optimization, delivering more adaptive models and solutions for heterogeneous data. This evolution will cater to increasingly complex and diverse collaborative computing scenarios.

Supplementary Note 3

The procedures of sharing tasks based on `MatSwarm`

We use the training of perovskite formation energy as an example to demonstrate the entire training process on `MatSwarm`.

Task submission.

As shown in Supplementary Fig. 3

Step ①: The task issuer inputs the relevant information of the task on the platform's front end and selects the training dataset. This step includes an option to enable the Trusted Execution Environment (TEE). When TEE is enabled, the smart contracts for the aggregation process of all models will execute within the TEE enclave environment, ensuring the confidentiality and integrity of the aggregation process.

Step ②: After submitting the task information, the platform's web server issues the training task and broadcasts it via the blockchain to the invitees selected by the task issuer.

Step ③: Upon receiving the task, the invitees can choose to accept or decline it through the blockchain network via the front-end interface and select their training datasets.

Step ④: When the task issuer receives more than two acceptance messages within a specified time interval, a task channel is created, and the aggregation smart contracts are deployed.

Step ⑤: The nodes that accepted the invitation join the task channel, preparing to execute the training task.

By following these steps, `MatSwarm` ensures a secure, efficient, and collaborative training process, making it easier for different organizations to work together on predicting new material properties without sharing raw data.

Task execution.

As shown in Supplementary Fig. 4:

Step ①: After the task issuer clicks the "Start Training" button on the front-end, the instruction is sent to the webserver, which broadcasts the command to all invitees via the blockchain.

Step ②: The selected training datasets are retrieved from the materials database and called to the webserver.

Step ③: Each participant uses the local model to train on their respective datasets.

Step ④: After each round of local model training, the aggregation smart contracts are invoked to aggregate the local model parameters. The local models of all participants are updated with the aggregated global parameters. This iterative process continues for 200 rounds, after which the training is stopped, resulting in a trained global model. The trained global model is then recorded on the blockchain, allowing any authorized organization to use this model to predict the formation energy of perovskite.

Supplementary Note 4

Description of Features Engineering

Feature Engineering in Experiments. The perovskite dataset used in predicting the formation energy, consists of 3967 samples. From these samples, 15 features were derived through sampling, as shown in Supplementary Table 2.

Feature engineering is crucial for predicting the formation energy of perovskites. Different feature engineering methods can extract various aspects of material characteristics, thereby enhancing the predictive capability of machine learning models. Each participant can select appropriate feature engineering methods based on the sampling characteristics of their material datasets, utilizing Matminer's feature engineering methods [33]. In this experiment, we employed the following four feature engineering methods for our perovskite dataset:

1. *StrtoComposition:* This method converts structural information into chemical composition information, transforming descriptions of crystal structures (such as chemical formulas or atomic arrangements) into representations of constituent elements and their proportions. This enables the model to utilize chemical composition information for predictions. The feature obtained from this method is *composition*.
2. *ElementProperty:* This method extracts the intrinsic properties of each element. Features are generated based on the properties of the elements in the material (such as atomic radius, electronegativity, ionization energy, etc.). These properties reflect the chemical and physical characteristics of the elements and are crucial for predicting perovskite formation energy. Among them, the Magpie features include not only the properties of individual elements but also their statistical information, such as mean, standard deviation, maximum, and minimum. These statistics provide a comprehensive reflection of the overall characteristics of the material, enhancing the model's predictive ability. This method yields 132 features, details of which can be found in the *Supplementary Material of the perovskite dataset in our public repository*.
3. *CompositionToOxidComposition:* This method converts chemical compositions into oxidation state compositions. By decomposing the elements in the chemical composition into different oxidation states, new features are generated. This helps the model understand the redox properties of the material, providing richer information for predicting formation energy. The feature obtained from this method is *composition_oxid*.
4. *OxidationStates:* This method extracts the oxidation state information of elements. The oxidation state of an element is a critical factor influencing its chemical reactivity and crystal structure stability, significantly affecting the formation energy of perovskites. The features obtained from this method are *minimum oxidation state, maximum oxidation state, range oxidation state, and std_dev oxidation state*.

Each feature engineering method has its unique role. By extracting information from different layers and aspects, these methods enrich the input features of the model, thereby improving prediction accuracy. Using these methods allows

for a more comprehensive capture of the chemical and physical characteristics of materials, providing a solid foundation for predicting formation energy.

Feature Importance Analysis Using SHAP Values. `MatSwarm` provides an efficient and secure solution for collaborative material performance prediction using data from multiple organizations while protecting the privacy of each participant's data. After users submit collaborative computing tasks on the platform, the task progress can be monitored in real-time through the task list. The task status includes information such as training time, number of training rounds, and the current round's training results. Upon task completion, each participant receives the predicted results and necessary data analysis, including MSE, R^2, RMSE, MAE, a scatter plot of the formation energy prediction results, a loss convergence curve, and a feature importance analysis chart. In the experiment of perovskite formation energy prediction in this paper, part of the returned results are shown in Supplementary Fig. 5.

Supplementary Fig. 5 illustrates the importance of the above features for the perovskite formation energy prediction model. In feature importance analysis, SHapley Additive exPlanations (SHAP) [34] is a game theory-based method used to explain the output of machine learning models. SHAP values provide a consistent and fair way to allocate each feature's contribution to the prediction results. Supplementary Fig. 5-(a) displays the top 20 most important features for the model. Supplementary Fig. 5-(b) shows the SHAP summary plot for the MLP regression model. The Y-axis represents the feature names, arranged from top to bottom in descending order of importance. The SHAP values corresponding to the features are shown on the X-axis, indicating the impact of a feature on the model output. For details about the implementation process and results, please refer to *Supplementary Movie 3*.

Supplementary Note 5

Adaptability of `MatSwarm` in Materials Science and Beyond

The `MatSwarm` framework is specifically designed to address challenges within the materials science domain. Its primary objective is to solve material-related regression problems in situations where sharing raw data is not possible. The framework achieves this by enabling secure collaborative computing, allowing organizations to leverage their private datasets without exposing sensitive information. Although `MatSwarm` is tailored for materials science, its design principles and methodologies offer valuable insights and potential applications for other domains with similar data-sharing constraints. This section provides a comprehensive guide on how to extend the `MatSwarm` framework beyond the materials science domain, offering methodologies and strategies for applying it to other domains with similar data-sharing challenges.

Generalization Within the Materials Science Domain. In the materials science domain, `MatSwarm` demonstrates its adaptability through the application of consistent methodologies across various datasets and scientific problems. `MatSwarm` can effectively tackle diverse regression problems, from predicting material properties to addressing other collaborative computation problems such as crystal structure optimization, failure model identification, and new material development. As shown in Supplementary Fig. 6, Organizations can initiate various shared tasks in the field of materials on the platform, such as predicting perovskite formation energy, forecasting the elastic properties of silicon materials, and optimizing the microstructure of high-performance alloys. This adaptability allows the framework to provide generalized solutions within materials science and offers a reference for extending its application to other domains.

Extending `MatSwarm` to Other Domains. Although `MatSwarm` is tailored for the materials science domain, its data-driven methodologies provide a valuable template for addressing similar challenges in other domains. By leveraging its robust modular design principles, organizations in these domains can customize core components of `MatSwarm`—including the objective function, dataset selection, local models, aggregation methods, and output results—to address challenges similar to those found in materials science. This modular approach allows the framework to be adapted to meet specific application requirements, enabling it to effectively solve domain-specific problems while ensuring data security and maintaining robust model performance across diverse data distributions.

Customization and Extensibility of Core Components

This subsection outlines the key components of the `MatSwarm` framework and how they can be tailored to suit various shared tasks. These core components include:

1. *Objective Function Design:* In the materials science domain, we primarily focus on regression problems, such as predicting material properties. For different

shared tasks, the objective function generally does not require further adjustment or customization. However, in other domains, the objective function can be tailored to meet the specific goals of a given task, whether it involves regression, classification, or clustering. Customizing the objective functions enables the framework to adapt to a wide range of applications across various domains.

2. *Dataset Selection:* `MatSwarm` supports the integration of heterogeneous datasets, allowing users to select datasets that best represent their research questions or application areas. This capability ensures that the framework can handle various data types and sources. As shown in Supplementary Fig. 7, each organization can view all datasets to which it has access on the NMDMS platform. For example, when initiating a shared task for predicting perovskite formation energy, organizations can search for "perovskite" within their datasets to select the dataset for training.
3. *Local Model Selection:* `MatSwarm` provides a library of local models that can be chosen based on the shared task. Users can select models that best fit the data characteristics and task objectives. As shown in Supplementary Fig. 8, the platform currently offers local models such as MLP, Lasso, LSTM, RNN, and CNN. For predicting perovskite formation energy, MLP is verified to be a suitable model for this training task. The method provided here can be extended according to specific training tasks to meet the actual needs of users to the greatest extent.
4. *Aggregation Method Selection:* The aggregation methods within `MatSwarm` are designed to be adaptable based on the shared task, including considerations for Byzantine resilience and specific performance goals. As shown in Supplementary Fig. 8, the platform currently offers aggregation methods such as Mean, Median, MultiKrum, CenteredClipping, and GeoMed. For predicting perovskite formation energy, Mean is verified to be a suitable model for this training task. The method provided here can be extended according to specific training tasks to meet the actual needs of users to the greatest extent.
5. *Output Results Customization:* `MatSwarm` offers flexibility in selecting output formats, such as scatter plots, loss convergence curves, or SHAP value bar charts, depending on the task requirements. As shown in Supplementary Fig. 8, the platform currently offers output result graphs such as scatter plot of predicted values, loss convergence curve, SHAP value bar chart, and MSE curve. Assuming we selected the scatter plot of predicted values and the loss convergence curve, as shown in Supplementary Fig. 9, the results presented upon the completion of the shared task correspond to all the key components we selected. The presentation methods and forms provided here can be extended according to specific training tasks to meet the actual needs of users to the greatest extent.

By allowing users to tailor core elements such as objective functions, datasets, local models, aggregation methods, and output formats, `MatSwarm` can effectively adapt to a wide range of research and application needs. This adaptability ensures that `MatSwarm` is not only a powerful tool for the materials science domain but also a template for other domains requiring collaborative computation and data-driven

insights. Based on the minimal open-sourced system we provide, users can customize their own platform applications according to practical needs.

Considerations for Applying `MatSwarm` to Other Domains

When considering the application of `MatSwarm` to other domains, the following factors should be taken into account:

1. *Application Requirements:* `MatSwarm` is particularly well-suited for domains that require collaborative computation and data-driven insights, especially in scenarios where there is a strict requirement for the protection of sensitive data. The framework's adaptability makes it ideal for tasks involving regression problems while ensuring data privacy and security. Furthermore, effective material development often requires multi-institutional collaboration, which `MatSwarm` facilitates by enabling swarm learning. This allows institutions to collaboratively build a shared model, benefiting from pooled data while respecting each organization's data privacy.
   - *Sensitive Data:* Materials data often contains sensitive or proprietary information that must be protected. `MatSwarm` uses secure computation techniques like Trusted Execution Environments (TEE) to ensure data confidentiality during model training and aggregation.
   - *Collaboration Needs:* Due to strict regulation constraints, data is often siloed within organizations, limiting collaboration. The framework allows for secure data sharing and model training without exposing raw data, fostering cooperation while maintaining privacy.

   Domains beyond materials science that require both sensitive data protection and collaborative computation can reference the design and implementation methods of the `MatSwarm` framework. For instance, in the healthcare sector, where patient privacy is crucial, the framework can serve as a model for developing secure collaborative analytics platforms. Similarly, in finance, where proprietary data must be safeguarded, `MatSwarm`'s approach to secure computation can be adapted to enable shared insights without exposing raw data. Leveraging its modular design principles and secure computation techniques, this framework can be adapted to other fields to address their specific challenges, incorporating domain-specific knowledge. This approach facilitates data-driven collaboration while ensuring the confidentiality of sensitive data.
2. *Dataset Preprocessing:* In the materials science domain, data often exhibits non-independent and identically distributed (non-i.i.d.) characteristics due to variations in material sources, testing equipment, and methodologies. To address this heterogeneity, this paper presents a swarm transfer learning method specifically designed to improve model accuracy on such data. Our experimental results demonstrate that the method maintains high accuracy even when data is i.i.d. Furthermore, a key advantage of this research is the platform's ability to store datasets from all organizations in a standardized format using dynamic

containers [32]. This provides a solid foundation for collaborative computing across organizations, allowing them to use private datasets stored on the platform without requiring pre-processing.

In applying this framework to other domains, organizations will need to engage in data pre-processing, such as data cleaning, standardization, and alignment. However, the distribution characteristics of datasets between organizations are not a primary concern in the framework's usage. Whether the data is i.i.d. or non-i.i.d., the swarm transfer learning method described in this paper can be employed for model training.

3. *System Requirements:* The `MatSwarm` framework is designed with a robust infrastructure that facilitates secure and efficient collaborative computation. At its core, `MatSwarm` integrates several key components to ensure data privacy, integrity, and computational efficiency. The framework employs a blockchain-based network implemented using Hyperledger Fabric to manage secure data sharing and provenance tracking. This allows participants to exchange information without exposing raw data. Additionally, for computations that require heightened confidentiality and integrity, `MatSwarm` utilizes Trusted Execution Environments (TEEs) powered by Intel SGX. This infrastructure ensures that sensitive data remains protected throughout the computation process, providing a secure environment for executing swarm learning algorithms.

   Other domains looking to implement a similar framework should have the necessary infrastructure to support `MatSwarm`'s computational demands. This includes building a blockchain network for secure data sharing and, if there are high requirements for confidentiality and integrity during swarm learning computations, constructing a confidential computing environment using Trusted Execution Environments (TEEs), such as Intel SGX, ARM TrustZone, etc.
4. *Customization Needs:* In the materials science domain, `MatSwarm` is primarily used to solve regression problems, providing a versatile solution for various materials-related regression tasks. To accommodate the diverse nature of these tasks, the platform is designed with a modular approach, allowing for flexibility and adaptability. Local models, aggregation methods, and output results can be selected based on the specific requirements of each task, and the system supports expansion and updates to incorporate new methods and models as needed.

   When applying this framework to other domains, organizations should be prepared to customize `MatSwarm`'s components to fit the specific needs of their domain, which may include a variety of tasks beyond regression. This includes tailoring the five core components: *objective functions, datasets, local models, aggregation methods, and output results*. Specific details on customizing these core components will be provided in the following subsection to ensure that the framework is tailored to address unique challenges and goals effectively.

Supplementary Figures

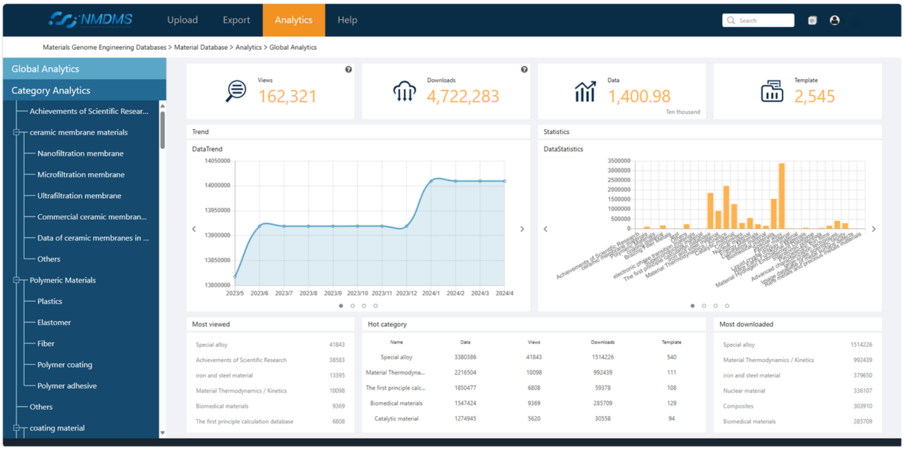

Supplementary Fig. 1: Serving as foundational support for MGE data applications, NMDMS has successfully accumulated over 14 million pieces of valid material data, and more information could be referred to [31, 32].

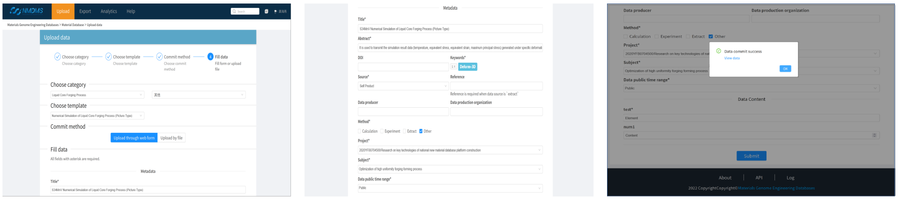

(a) (b) (c)

Supplementary Fig. 2: Graphical user interface for uploading data. (a) Select the corresponding category and template. (b) Fill in the metadata. (c) Fill in the original data.

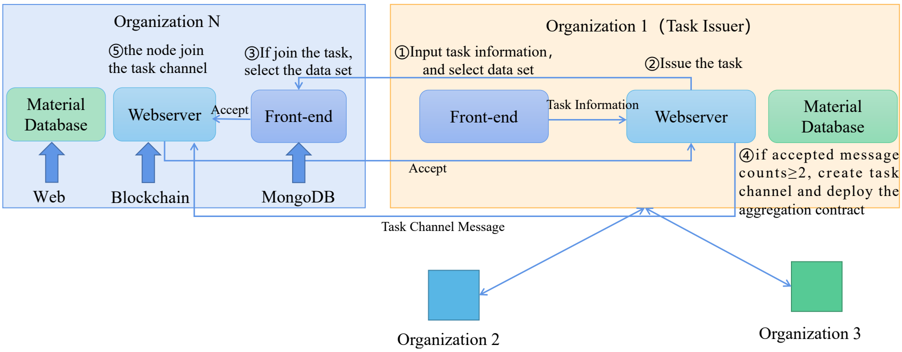

Supplementary Fig. 3: The procedures of `MatSwarm` task submission. Steps ①-⑤ are detailed in Supplementary Note 3.

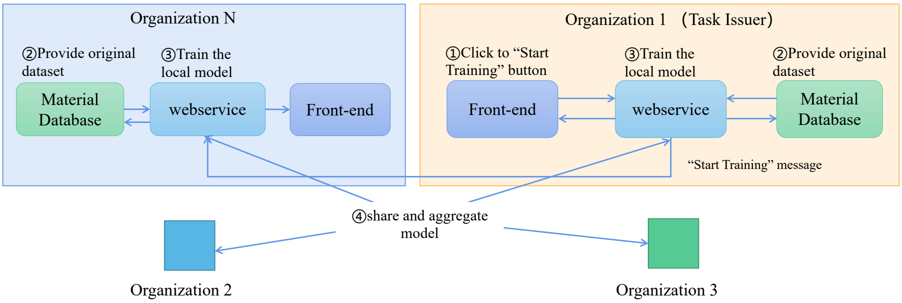

Supplementary Fig. 4: The procedures of `MatSwarm` task execution. Steps ①-④ are detailed in Supplementary Note 3.

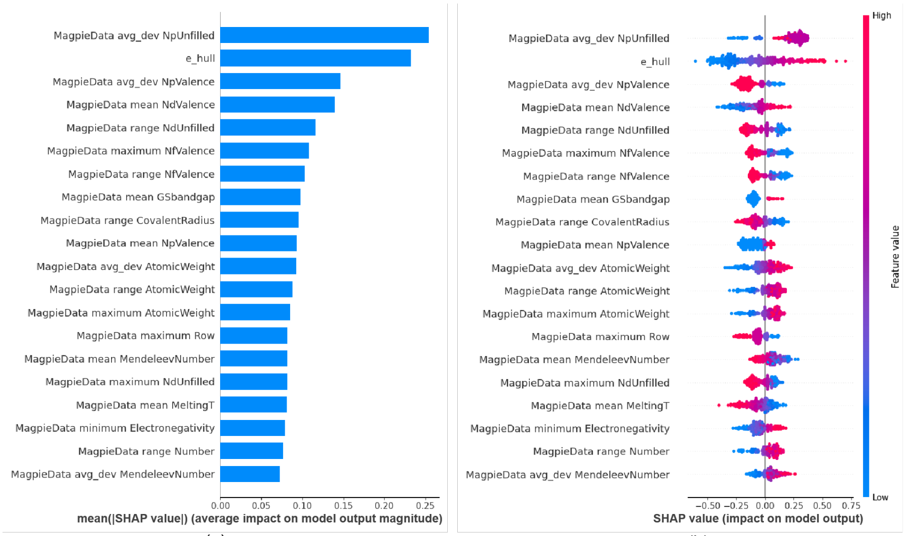

(a) (b)

Supplementary Fig. 5: Feature importance graph for the MLP regression model. Features at the top play the most important role in predicting the formation energy. (a) Average impact on model output magnitude. (b) The values of a certain feature influence the formation energy. In the summary plot, each point represents a specific instance (row) of the corresponding feature. The color of the points represents the SHAP value of the corresponding feature, with high values in red and low values in blue. A positive SHAP value indicates that the feature increases the predicted value, while a negative SHAP value indicates that the feature decreases the predicted value.

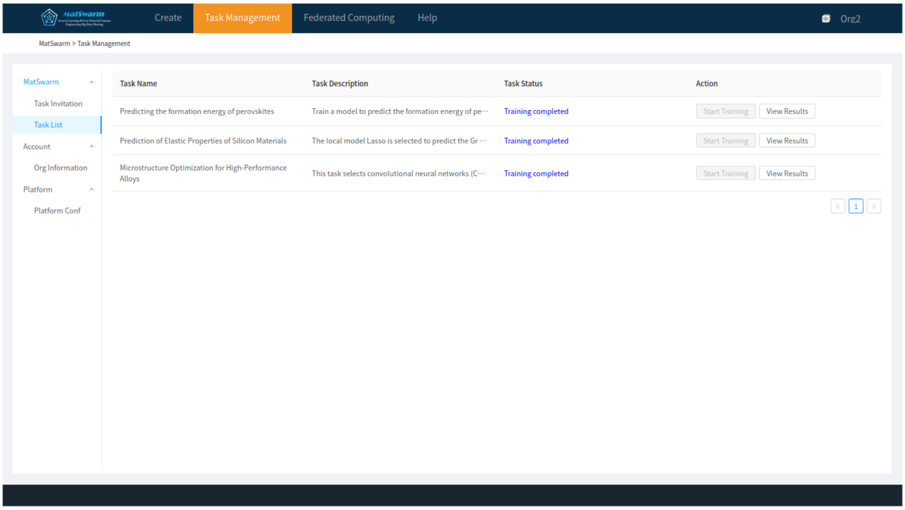

Supplementary Fig. 6: The screenshot of a shared task list. Organizations can initiate various shared tasks in the field of materials on the platform.

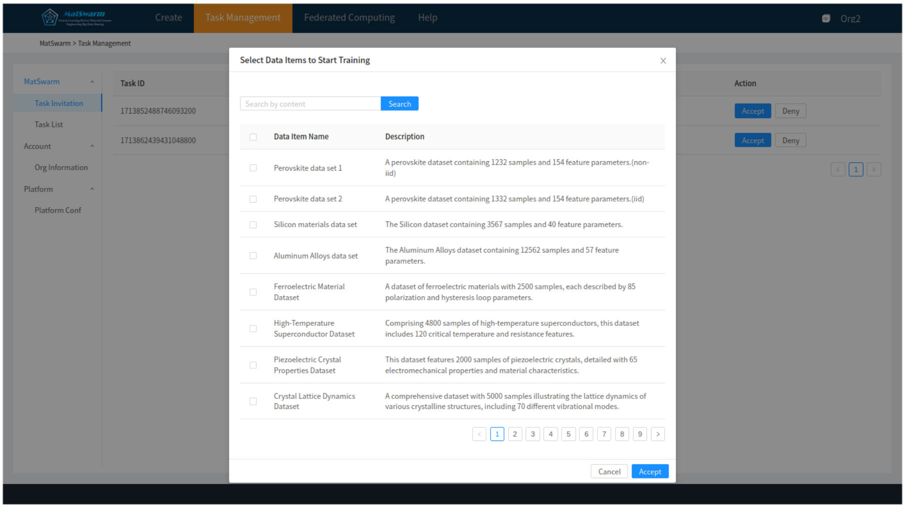

(a)

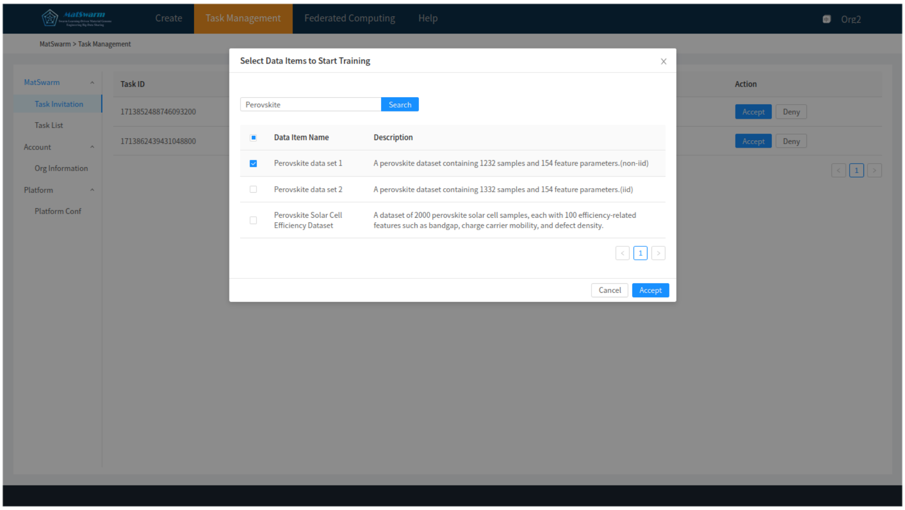

(b)

Supplementary Fig. 7: The screenshot of dataset selection. (a) All local datasets of Organization 2. (b) Datasets available for selection after searching for "perovskite".

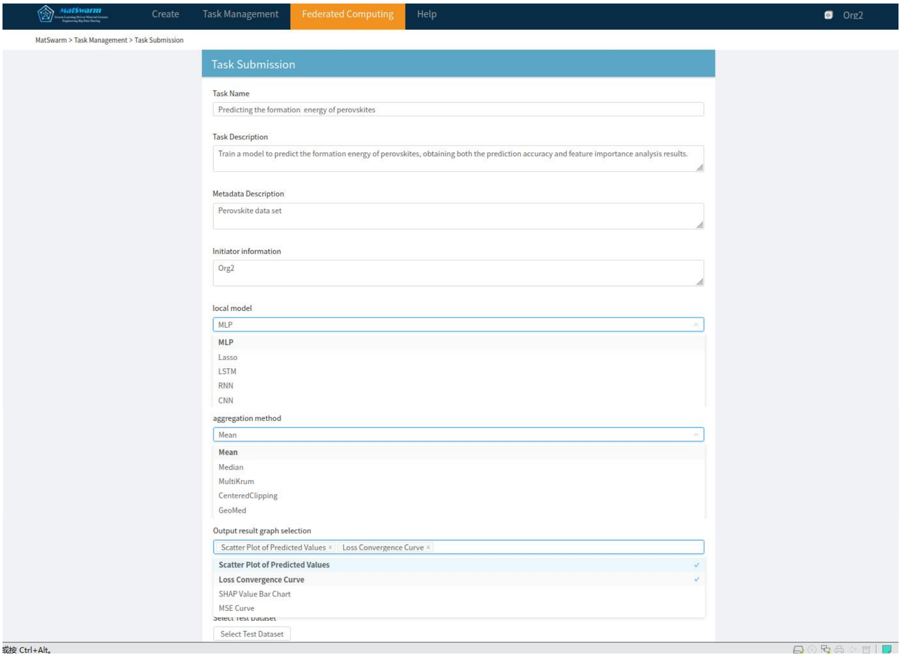

Supplementary Fig. 8: The screenshot of selecting key components in a shared task. The platform currently offers aggregation methods such as Mean, Median, Multi-Krum, CenteredClipping, and GeoMed. Also, it offers output result graphs such as scatter plot of predicted values, loss convergence curve, SHAP value bar chart, and MSE curve.

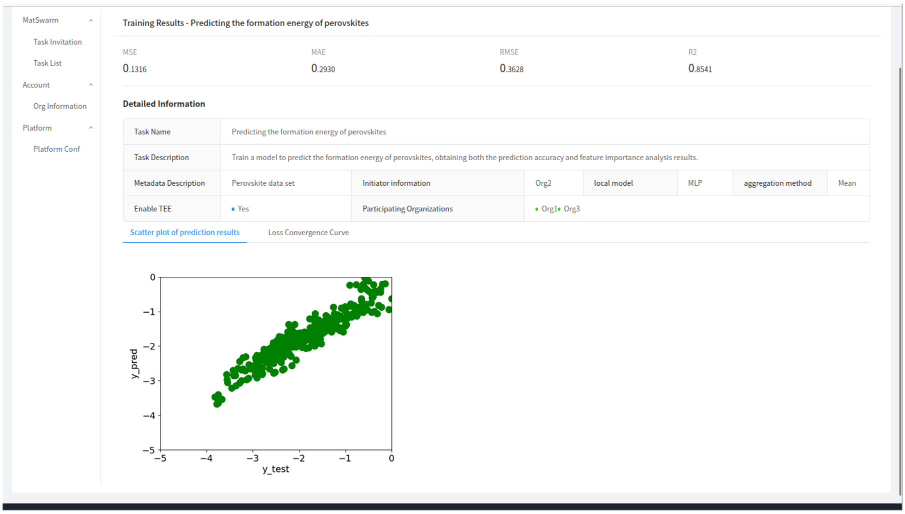

Supplementary Fig. 9: The screenshot of a shared task result. These results presented upon the completion of the shared task correspond to all the key components users selected.

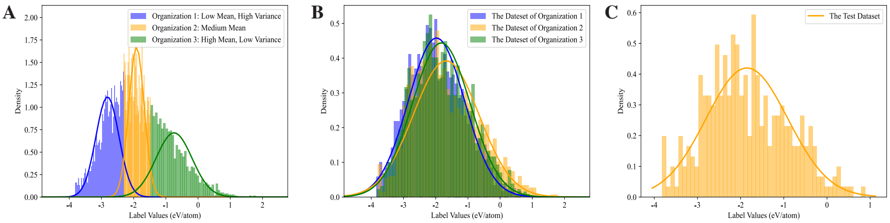

Supplementary Fig. 10: Distribution of data set labels for the three participants: (a) non-i.i.d.(non-independent and identically distributed) for training, We sorted the samples by their label values in ascending order, then evenly divided them into three datasets to create significant differences in their means and variances. (b) i.i.d.(independent and identically distributed) for training, whose variances and means are substantially similar. (c) test data set, whose data label distribution is consistent with the label distribution for the i.i.d. data set. The total number of samples in the training dataset is $n = 3694$, and the test set consists of 322 samples. The curve is a Gaussian fit to the data, illustrating the distribution of formation energy.

Supplementary Tables

Supplementary Table 1: The comparison of related work.

Framework	Description	Remark
Pokhrel et al. [22]	A blockchain-based autonomous federated learning design for privacy awareness and efficient vehicle network communication.	The computational cost is high, increasing the overall delay time.
Warnat et al. [23]	A swarm learning framework based on private computing technology for collaborative training of disease data, performing classification tasks while protecting patients' private data.	This method does not address the issue of heterogeneous medical data.
Saldanha et al. [24]	A swarm learning framework for training distributed AI models for histopathology image analysis tasks, eliminating the need for data transfer.	This framework faces the risk of model parameter leakage and does not account for data heterogeneity.
Wei et al. [27]	A framework based on Differential Privacy (DP) to prevent information leakage by adding artificial noise at the client-side before model aggregation, known as Noising before Aggregation Federated Learning (NbAFL).	Model aggregation is performed on centralized servers with low accuracy and efficiency.
Liu et al. [28]	The framework combines Homomorphic Encryption with Federated Transfer Learning to ensure the security of model parameters.	The computational demands of Homomorphic Encryption lead to poor prediction efficiency.
Yin et al. [35]	A hybrid privacy-preserving method that enhances the adaptability of different distributed datasets through local Bayesian differential privacy and a noise mechanism.	Model aggregation is performed on centralized servers with low model accuracy.
Kim et al. [36]	A blockchained federated learning (BlockFL) architecture where local learning model updates are exchanged and verified.	The model training and prediction process has poor security, and the deployment of blockchain on edge devices affect the overall efficiency of the framework.

Continued on next page.

–Continued from previous page.

**Framework**	**Description**	**Remark**
Qu et al. [37]	The blockchain-based federated learning (FL-Block) scheme enables autonomous machine learning to maintain the global model, and it coordinates using the Proof-of-Work consensus mechanism of the blockchain.	The model aggregation process is prone to tampering attacks, and the consensus mechanism is low efficient.
Kalapaaking et al. [38]	A blockchain-based Federated Learning framework with Intel SGX-based Trusted Execution Environment for securely aggregating local models in the Industrial Internet-of-Things.	This framework cannot effectively resist Byzantine node disruptions during model aggregation.
MatSwarm	A novel collaborative model training framework designed specifically for the material science domain.	It provides an efficient and highly accurate swarm learning framework for joint model prediction by material organizations, ensuring data security throughout the entire model lifecycle.

Supplementary Table 2: Features and their descriptions.

**Feature Symbol**	**Description**
atom A	The atom in the ‘A’ site
atom B	The atom in the ‘B’ site
a	Lattice parameter a
b	Lattice parameter b
c	Lattice parameter c
alpha	Lattice angle alpha
beta	Lattice angle beta
gamma	Lattice angle gamma
bandgap	Bandgap in eV from PBE calculations
lowest distortion	Local distortion crystal structure with lowest energy among all considered distortions
vpa	Volume per atom (Å^3/atom)
mub	Magnetic moment
eform	Formation energy in eV
eform oxygen	Formation energy of oxygen vacancy (eV)
e_hull	Energy above convex hull (eV)

References

[1] J. Wen, Z. Zhang, Y. Lan, Z. Cui, J. Cai, W. Zhang, A survey on federated learning: challenges and applications. International Journal of Machine Learning and Cybernetics **14**(2), 513–535 (2023)

[2] Q. Yang, Y. Liu, T. Chen, Y. Tong, Federated machine learning: Concept and applications. ACM Transactions on Intelligent Systems and Technology (TIST) **10**(2), 1–19 (2019)

[3] H. Zhu, J. Xu, S. Liu, Y. Jin, Federated learning on non-iid data: A survey. Neurocomputing **465**, 371–390 (2021)

[4] B. McMahan, E. Moore, D. Ramage, S. Hampson, B.A. y Arcas, Communication-efficient learning of deep networks from decentralized data. Artificial intelligence and statistics pp. 1273–1282 (2017)

[5] Q. Li, B. He, D. Song, Model-contrastive federated learning. Proceedings of the IEEE/CVF Conference on Computer Vision and Pattern Recognition (CVPR) pp. 10,713–10,722 (2021)

[6] T. Li, A.K. Sahu, M. Zaheer, M. Sanjabi, A. Talwalkar, V. Smith, Federated optimization in heterogeneous networks. Proceedings of Machine learning and systems **2**, 429–450 (2020)

[7] N. Yoshida, T. Nishio, M. Morikura, K. Yamamoto, R. Yonetani, Hybrid-fl: Cooperative learning mechanism using non-iid data in wireless networks. CoRR **abs/1905.07210** (2019)

[8] M.A. Tanner, W.H. Wong, The calculation of posterior distributions by data augmentation. Journal of the American statistical Association **82**(398), 528–540 (1987)

[9] Y. Kim, Y. Kim, C. Yang, K. Park, G.X. Gu, S. Ryu, Deep learning framework for material design space exploration using active transfer learning and data augmentation. npj Computational Materials **7**(1), 140 (2021)

[10] S. Jain, G. Seth, A. Paruthi, U. Soni, G. Kumar, Synthetic data augmentation for surface defect detection and classification using deep learning. Journal of Intelligent Manufacturing **33**, 1007–1020 (2022)

[11] S.P. Karimireddy, S. Kale, M. Mohri, S.J. Reddi, S.U. Stich, A.T. Suresh, Scaffold: Stochastic controlled averaging for federated learning. Proceedings of the 37th International Conference on Machine Learning **119**, 5132–5143 (2020)

[12] L. Gao, H. Fu, L. Li, Y. Chen, M. Xu, C.Z. Xu, Feddc: Federated learning with non-iid data via local drift decoupling and correction. Proceedings of the

IEEE/CVF conference on computer vision and pattern recognition pp. 10,112–10,121 (2022)

[13] J. Gou, B. Yu, S.J. Maybank, D. Tao, Knowledge distillation: A survey. International Journal of Computer Vision **129**(6), 1789–1819 (2021)

[14] C. Bucilua, R. Caruana, A. Niculescu-Mizil, Model compression. Proceedings of the 12th ACM SIGKDD International Conference on Knowledge Discovery and Data Mining pp. 535–541 (2006)

[15] L. Zhang, L. Shen, L. Ding, D. Tao, L.Y. Duan, Fine-tuning global model via data-free knowledge distillation for non-iid federated learning. Proceedings of the IEEE/CVF Conference on Computer Vision and Pattern Recognition (CVPR) pp. 10,174–10,183 (2022)

[16] H. Guo, X. Yu, A survey on blockchain technology and its security. Blockchain: research and applications **3**(2), 100,067 (2022)

[17] Z. Chen, W. Xu, B. Wang, H. Yu, A blockchain-based preserving and sharing system for medical data privacy. Future Generation Computer Systems **124**, 338–350 (2021)

[18] K. Fan, Q. Pan, K. Zhang, Y. Bai, S. Sun, H. Li, Y. Yang, A Secure and Verifiable Data Sharing Scheme Based on Blockchain in Vehicular Social Networks. IEEE Transactions on Vehicular Technology **69**(6), 5826–5835 (2020)

[19] D.C. Nguyen, M. Ding, Q.V. Pham, P.N. Pathirana, L.B. Le, A. Seneviratne, J. Li, D. Niyato, H.V. Poor, Federated learning meets blockchain in edge computing: Opportunities and challenges. IEEE Internet of Things Journal **8**(16), 12,806–12,825 (2021)

[20] Y. Zhao, J. Zhao, L. Jiang, R. Tan, D. Niyato, Z. Li, L. Lyu, Y. Liu, Privacy-preserving blockchain-based federated learning for iot devices. IEEE Internet of Things Journal **8**(3), 1817–1829 (2020)

[21] M. Shayan, C. Fung, C.J. Yoon, I. Beschastnikh, Biscotti: A blockchain system for private and secure federated learning. IEEE Transactions on Parallel and Distributed Systems **32**(7), 1513–1525 (2020)

[22] S.R. Pokhrel, J. Choi, Federated Learning With Blockchain for Autonomous Vehicles: Analysis and Design Challenges. IEEE Transactions on Communications **68**(8), 4734–4746 (2020)

[23] S. Warnat-Herresthal, H. Schultze, K.L. Shastry, S. Manamohan, S. Mukherjee, V. Garg, R. Sarveswara, K. Händler, P. Pickkers, N.A. Aziz, et al., Swarm learning for decentralized and confidential clinical machine learning. Nature **594**(7862), 265–270 (2021)

[24] O.L. Saldanha, P. Quirke, N.P. West, J.A. James, M.B. Loughrey, H.I. Grabsch, M. Salto-Tellez, E. Alwers, D. Cifci, N. Ghaffari Laleh, et al., Swarm learning for decentralized artificial intelligence in cancer histopathology. Nature medicine **28**(6), 1232–1239 (2022)

[25] K. Wei, J. Li, M. Ding, C. Ma, H.H. Yang, F. Farokhi, S. Jin, T.Q. Quek, H.V. Poor, Federated learning with differential privacy: Algorithms and performance analysis. IEEE Transactions on Information Forensics and Security **15**, 3454–3469 (2020)

[26] J. Ma, S.A. Naas, S. Sigg, X. Lyu, Privacy-preserving federated learning based on multi-key homomorphic encryption. International Journal of Intelligent Systems **37**(9), 5880–5901 (2022)

[27] K. Wei, J. Li, M. Ding, C. Ma, H.H. Yang, F. Farokhi, S. Jin, T.Q.S. Quek, H.V. Poor, Federated Learning With Differential Privacy: Algorithms and Performance Analysis. IEEE Transactions on Information Forensics and Security **15**, 3454–3469 (2020)

[28] Y. Liu, Y. Kang, C. Xing, T. Chen, Q. Yang, Secure Federated Transfer Learning. IEEE Intelligent Systems **35**(4), 70–82 (2020)

[29] A. El Ouadrhiri, A. Abdelhadi, Differential privacy for deep and federated learning: A survey. IEEE access **10**, 22,359–22,380 (2022)

[30] D. Lee, D. Kohlbrenner, S. Shinde, K. Asanović, D. Song, Keystone: An open framework for architecting trusted execution environments. Proceedings of the Fifteenth European Conference on Computer Systems pp. 1–16 (2020)

[31] R. Wang, C. Xu, R. Dong, Z. Luo, R. Zheng, X. Zhang, A secured big-data sharing platform for materials genome engineering: State-of-the-art, challenges and architecture. Future Generation Computer Systems **142**, 59–74 (2023)

[32] S. Liu, Y. Su, H. Yin, D. Zhang, J. He, H. Huang, X. Jiang, X. Wang, H. Gong, Z. Li, et al., An infrastructure with user-centered presentation data model for integrated management of materials data and services. Npj Computational Materials **7**(1), 88 (2021)

[33] L. Ward, A. Faghaninia, A. Jain, M. Dunn. Matminer: An open-source toolkit for materials data mining (2018). URL https://hackingmaterials.lbl.gov/matminer/. Accessed: 2024-05-31

[34] M. Shen, J. Duan, L. Zhu, J. Zhang, X. Du, M. Guizani, Blockchain-based incentives for secure and collaborative data sharing in multiple clouds. IEEE Journal on Selected Areas in Communications **38**(6), 1229–1241 (2020)

[35] L. Yin, J. Feng, H. Xun, Z. Sun, X. Cheng, A privacy-preserving federated learning for multiparty data sharing in social iots. IEEE Transactions on Network Science and Engineering **8**(3), 2706–2718 (2021)

[36] H. Kim, J. Park, M. Bennis, S.L. Kim, Blockchained on-device federated learning. IEEE Communications Letters **24**(6), 1279–1283 (2019)

[37] Y. Qu, L. Gao, T.H. Luan, Y. Xiang, S. Yu, B. Li, G. Zheng, Decentralized privacy using blockchain-enabled federated learning in fog computing. IEEE Internet of Things Journal **7**(6), 5171–5183 (2020)

[38] A.P. Kalapaaking, I. Khalil, M.S. Rahman, M. Atiquzzaman, X. Yi, M. Almashor, Blockchain-based federated learning with secure aggregation in trusted execution environment for internet-of-things. IEEE Transactions on Industrial Informatics **19**(2), 1703–1714 (2022)
