# Supplementary material for: MatSwarm: trusted swarm transfer learning driven materials computation for secure big data sharing
Source: Nat Commun. 2024 Oct 28;15:9290. doi: 10.1038/s41467-024-53431-x (PMC11519480; doi:10.1038/s41467-024-53431-x)
Supplement: Supplementary file 3 — Description of Additional Supplementary Files [file 41467_2024_53431_MOESM3_ESM.docx]

**Description of Additional Supplementary Files**

**Supplementary Movie 1:** Introduction of MatSwarm

**Supplementary Movie 2:** MatSwarm-procedures

**Supplementary Movie 3:** MatSwarm-operations
